# ATP-sensitive inward rectifier potassium channels reveal functional linkage between salivary gland function and blood feeding in the mosquito, *Aedes aegypti*

**DOI:** 10.1038/s42003-022-03222-1

**Published:** 2022-03-28

**Authors:** Zhilin Li, Alexander Soohoo-Hui, Flinn M. O’Hara, Daniel R. Swale

**Affiliations:** Louisiana State University AgCenter, Department of Entomology, Baton Rouge, LA 70803 USA

**Keywords:** Entomology, Animal physiology

## Abstract

Reducing saliva secretions into the vertebrate host reduces feeding efficacy by most hematophagous arthropods. However, seminal studies suggested saliva is not a prerequisite for blood feeding in *Aedes aegypti*. To test this paradigm, we manually transected the salivary duct of female *A. aegypti* and an inability to salivate was correlated to an inability to imbibe blood. These data justified testing the relevance of inwardly rectifying potassium (Kir) channels in the *A. aegypti* salivary gland as an antifeedant target site. Pharmacological activation of ATP-gated Kir (K_ATP_) channels reduced the secretory activity of the salivary gland by 15-fold that led to near elimination of blood ingestion during feeding. The reduced salivation and feeding success nearly eliminated horizontal transmission and acquisition of Dengue virus-2 (DENV2). These data suggest mosquito salivation is a prerequisite for blood feeding and provide evidence that K_ATP_ channels are critical for salivation, feeding, and vector competency.

## Abstract (web summary)

The salivary gland of Aedes aegypti is needed for efficient blood feeding, and disruption of ATP-gated Kir channels prevents salivation and blood feeding in A. aegypti as well as horizontal transmission and acquisition of Dengue virus2.

## Introduction

Hematophagous, or blood feeding, arthropods are responsible for the transmission of pathogenic agents that account for 17% of all infectious diseases worldwide^1^. More specifically, mosquitoes are vectors of numerous human pathogens that cause diseases relevant to human health, such as the yellow fever mosquito *Aedes aegypti* that is the primary vector of pathogens known to cause emerging or reemerging diseases in humans, such as dengue, zika, chikungunya, and yellow fever^2,3^. Furthermore, areas with endemic arbovirus transmission shoulder substantial economic burdens, with annual estimated costs of $2.1 billion in the Americas^4^ and nearly $1 billion in Southeast Asia^5^. These facts have fueled a constant race to develop new materials and methods of arthropod control, which is time-sensitive because resistance to modern synthetic pesticides is known to evolve over a relatively short time period and a lack of effective control measures underlies disease outbreaks in humans^6–8^. Thus, there is a need to increase the repertoire for arbovirus control by supplementing existing control strategies with promising approaches that focus on interrupting the transmission and maintenance cycles of arboviruses.

Secreted saliva of mosquito contains a diverse cocktail of pharmacologically active components that possess immunomodulatory and anti-inflammatory properties as well as inhibit host hemostasis, which facilitates blood meal acquisition^9^. Further, saliva potentiates arbovirus infection in mammalian models due to inhibition of host immune responses^10–13^. The critical role of mosquito saliva in blood meal acquisition and horizontal transmission of pathogens has stimulated attempts to develop vaccines against proteins in the secreted saliva^14–16^, which would hinder the development of the microenvironment required for pathogen dissemination in the host and systemic infection^16^. Yet, despite proof-of-concept studies in arthropod vectors and the advancements in the field of mosquito sialo-transcriptomics^9,17,18^, significant barriers to the development of mosquito saliva-directed vaccines remain and limit commercialization. For instance, differential responses by pathogen-vector pairs that cause increased pathogen transmission and disease manifestation, short-lived immune responses in human skin without ongoing exposure to mosquitoes, and the high specificity for pathogen or vector species reduces field efficacy. The majority of these barriers are likely to be eliminated by the development of products that inhibit salivary gland function because the physiological underpinnings of gland function are generally conserved across taxa^19^ and would not rely upon host immune responses. Thus, salivary gland-directed chemicals or vaccines would eliminate vector or pathogen specificity, which has been a problem for commercialization of tick vaccines^20,21^, and enable the development of pan-arthropod control measures^22,23^. However, seminal studies suggested saliva is not a prerequisite for mosquito blood-feeding^24–26^ and these findings curbed efforts to understand the mechanisms underlying mosquito salivary gland secretion and reduced enthusiasm for the development of salivary gland-directed molecules for mosquito antifeedants. Ultimately, our understanding of the functional underpinnings driving mosquito salivary gland function remains incomplete and this gap in knowledge has prevented further interrogation of the therapeutic potential of salivary gland-specific targets.

Potassium (K^+^) ions stand out as a compelling candidate for study regarding salivary gland function because they are the most abundant intracellular cation in vertebrates, invertebrates, and bacterial cells, which suggests perturbations of K^+^ ion flux will likely have deleterious consequences to tissue function. Although well studied in mammalian systems, the physiological relevance and toxicological potential of inward rectifier potassium (Kir) channels in insect systems have only recently begun to be realized^27^. These channels function as biological diodes in a variety of cell types and are critical for the maintenance of proper membrane potential and membrane resistance^28^, suggesting modulation of K^+^ flux through Kir channels will reduce cell and organ function. Indeed, our and others’ recent work has highlighted the critical role of inward rectifier potassium (Kir) channels in arthropod salivary gland function for hematophagous^29,30^ and sap^31–33^ feeding arthropods and further, recent reports have suggested flonicamid, a commercialized antifeedant insecticide, targets Kir channels to elicit the antifeedant activity^32^. Considering the growing evidence indicating salivary gland Kir channels are a poorly defined target for antifeedant insecticides, it is surprising that no information exists on the physiological contributions of Kir channels to mosquito feeding biology. Thus, the overarching goal of this investigation was to leverage multidisciplinary approaches to test the hypothesis that salivary gland function is required for successful mosquito blood-feeding and that blood meal ingestion and vector competency for dengue virus 2 in *A. aegypti* is reliant upon Kir channels expressed in the salivary gland. The data presented herein challenge existing paradigms by providing convincing evidence the mosquito salivary gland is a viable target tissue for therapeutic development to mitigate human health concerns and curb economic losses that result from mosquito feeding. Additionally, we provide the first evidence to our knowledge that ion channels, and specifically Kir channels, are critical for mosquito blood-feeding events and vector competency, which further establishes Kir channels as valid targets for the development of antifeedant insecticides.

## Results

### Transection of the salivary duct inhibits saliva secretion and blood-feeding

**Fig. 1:**
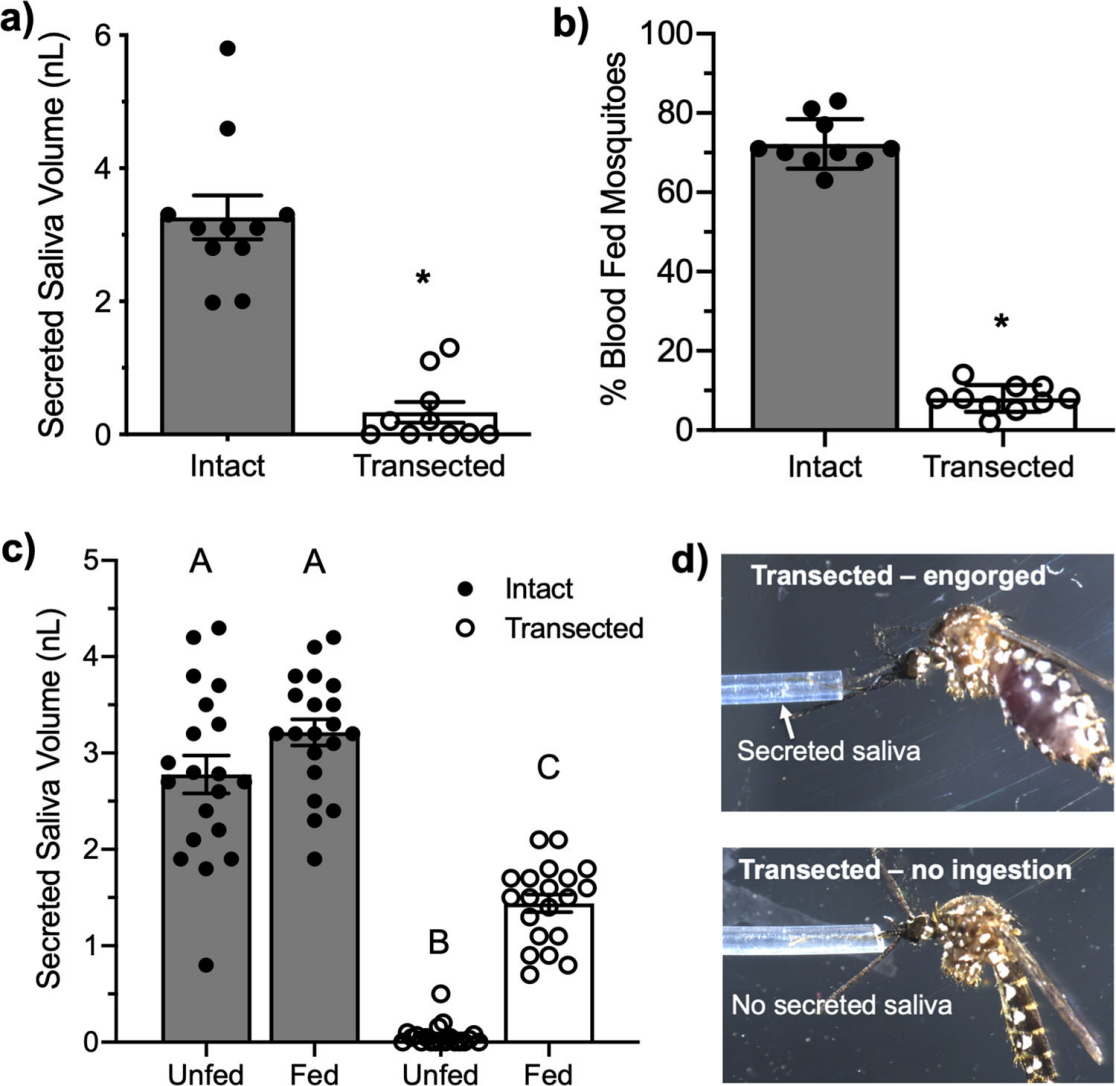
Relevance of salivary gland function to the blood-feeding ability of *A. aegypti*. **a** Secreted saliva from intact (control) or transected mosquitoes after DA exposure where bars represent mean (*n* = 5, 10 individuals per replicate) and error bars represent SEM. **b** Blood-feeding efficacy of *A. aegypti* after microsurgery to transect the salivary duct where bars represent mean (*n* = 10) and error bars represent SEM. For panels, **a**, **b**, asterisks represent statistical significance at *P* < 0.001 as determined by the unpaired *t*-test. **c** Secreted saliva of individual mosquitoes that have intact (closed circles, gray bars) or transected (open circles, white bars) salivary ducts. The mosquitoes studied in the transected group were the same transected individuals used in blood-feeding assays shown in panel **b** and the unfed represents the group that attempted to feed with no ingestion and the fed group represents engorged mosquitoes. Bars represent mean (*n* = 20 individuals) and error bars represent SEM. Bars not labeled by the same letter are significantly different from each other at *P* < 0.05 as determined by a one-way ANOVA with multiple comparisons posttest. **d** Representative images of saliva secretion from fed or unfed mosquitoes that underwent microdissection to transect the salivary duct showing blood-fed mosquitoes salivated (top panel) and no salivation from unfed mosquitoes (bottom panel). See Supplemental Data 1 for underlying data.

We aimed to validate previous claims that mosquitoes with transected salivary ducts were capable of blood-feeding to test if the salivary gland holds any potential for therapeutic development. Validation of transected ducts were performed through measurements of secreted saliva after injection with dopamine and transection of salivary ducts nearly eliminated saliva secretion with an average volume of 0.39 ± 0.32 nL, which is eightfold less saliva when compared to intact mosquitoes that secreted 3.1 ± 0.55 nL, a statistically significant reduction (*P* < 0.0001; Fig. 1a). A total of 13 ± 4% of mosquitoes that underwent microdissection to transect the salivary duct were capable of imbibing blood (Fig. 1b). We tested the secretory activity of these blood-fed mosquitos that underwent microdissection and interestingly, 100% of the mosquitoes that were blood-fed were capable of salivating with an average volume of 1.5 ± 0.7 nL (Fig. 1c). This volume of secreted saliva was approximately half the volume of intact blood-fed mosquitoes and statistically significant when compared to transected non-BF (*P* < 0.05) or fed intact (*P* < 0.05). Mosquitoes that underwent microdissection and repeatedly probed the membrane without obtaining a blood meal secreted 0.08 ± 0.05 nL of saliva (Fig. 1c). Importantly, the weighted average of the volume of secreted saliva from the transected blood-fed mosquitoes (13% of mosquitoes tested) and the secreted saliva of transected individuals that did not imbibe blood (87% of mosquitoes tested, Fig. 1c) is 0.27 nL, which is similar to the volume of secreted saliva from transected individuals (Fig. 1a). These data suggest the ability to imbibe blood after microdissection is likely due to incomplete transection of the duct or transection above the common salivary duct.

### Kir expression in *A. aegypti* salivary gland

**Fig. 2:**
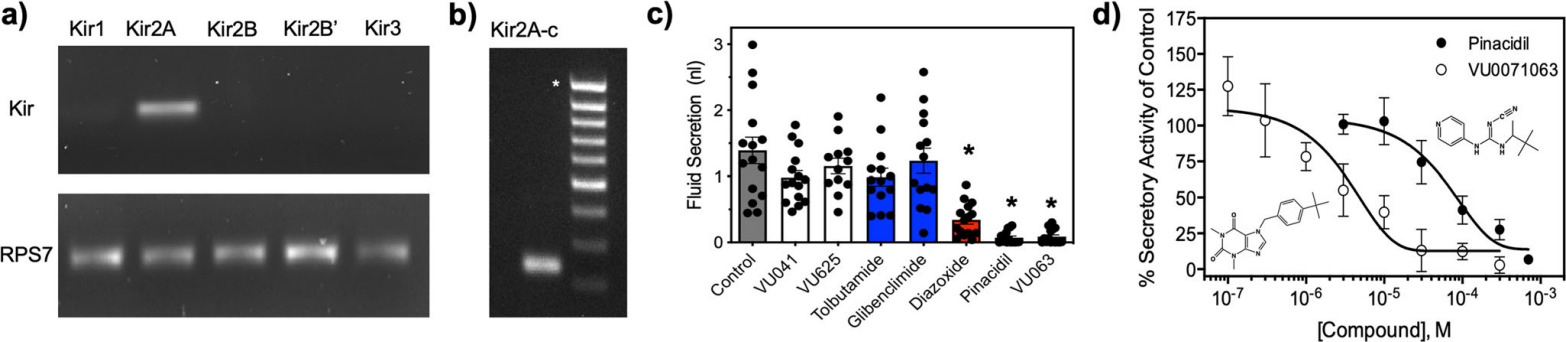
Influence of Kir channel modulators to saliva secretion. **a** Relative expression of *Ae*Kir channel genes in the salivary gland with the sole band representing cumulative *Ae*Kir2A expression (all splices) relative to RPS7. **b** Relative expression of *Ae*Kir2A splice variants in mosquito salivary glands as determined by semi-quantitative RT-PCR. Asterisk represents 1000 bp on the Thermo Scientific MassRuler DNA Ladder. **c** Total volume of secreted fluid after PBS injection (control) or after injection of Kir modulators. Bars represent mean (*n* = 15) volume secreted with error bars representing SEM. Statistical significance is denoted by the presence of an asterisk where *P < 0.05. **d** Concentration-response curve of pinacidil and VU0071063 influence to fluid secretion of adult female *A. aegypti*. Data points represent mean (*n* = 3) saliva secretion of control where each replicate consisted of 25 individuals. Insets show molecular structures of VU0071063 (left) and pinacidil (right). See Supplemental Data 2 for underlying data.

The indication that the intact salivary gland is in fact a prerequisite for mosquito blood-feeding justified testing the physiological and toxicological relevance of potassium ion conductance mechanisms in mosquito salivary glands. We characterized the expression of the five *Aedes aegypti* Kir channel genes in isolated salivary glands using qualitative RT-PCR. *A. aegypti* salivary glands express detectable levels of only Kir2A as indicated by the relative intensity of the bands (Fig. 2a and Supp. Fig. 1). The relative equal intensity of RPS7 bands across treatments indicates the differential Kir gene expression is not due to differences in gel loading techniques. *Aedes aegypti* Kir2A has three distinct splice variants, termed 2A-a. 2A-b, and 2A-c are 600 bp, 500 bp, and 250 bp, respectively^34^. These splice variants may constitute different conductance properties at the membrane and thus, we tested the expression of each splice variant in the salivary gland through PCR and data indicate Kir2A-c (band at 250 bp) is the dominant isoform in *A. aegypti* salivary glands and expression of other Kir2A splice variants are below detectable limits (Fig. 2b). To ensure the primer pairs used in this study can amplify other Kir encoding genes, we performed qRT-PCR with the midgut of *A. aegypti* and detected high-intensity bands for Kir1, Kir2A, Kir2B, and Kir3 (Supp. Fig. 2). These data are similar to previous reports that have shown weak expression of the Kir1 gene and high expression of Kir2A, Kir2B, and Kir3^34^.

### Saliva secretion is reduced by K_ATP_ activators, but not classical Kir inhibitors

To determine the influence Kir channel modulators have on the secretory activity of the salivary gland, we measured the total volume of saliva secreted after exposure to diverse Kir channel modulators through a modified fluid collection assay^35,36^. K_ATP_ channel activators, but not K_ATP_ inhibitors or Kir inhibitors, were found to inhibit the secretory activity of the mosquito gland. Control mosquitoes were shown to secrete an average of 1.2 ± 0.3 nL of saliva, which is similar to reported values^37^ and was not statistically significant when compared to VU041, VU625, tolbutamide, or glibenclamide (Fig. 2c). Pinacidil and VU0071063 were found to be equipotent in reducing saliva secretion and exhibited 15- and 13.3-fold less saliva secretion when compared to control mosquitoes (Fig. 2c). Diazoxide was less potent than pinacidil and VU0071063, but fluid secretion was still 3.3-fold reduced than control at the discriminatory concentration of 700 μM, a statistically significant (*P* < 0.05) reduction (Fig. 2c). Pinacidil and VU0071063 mediated inhibition of secretory activity was concentration-dependent with IC_50_ values of 79 μM (95% CI: 17-180; Hillslope: 0.86; *r*^2^: 0.75) and 630 nM (95% CI: 119–3652 nM; Hillslope: 0.55; *r*^2^: 0.9), respectively (Fig. 2d).

### K_ATP_ activators reduce blood meal ingestion by *Aedes aegypti*

**Fig. 3:**
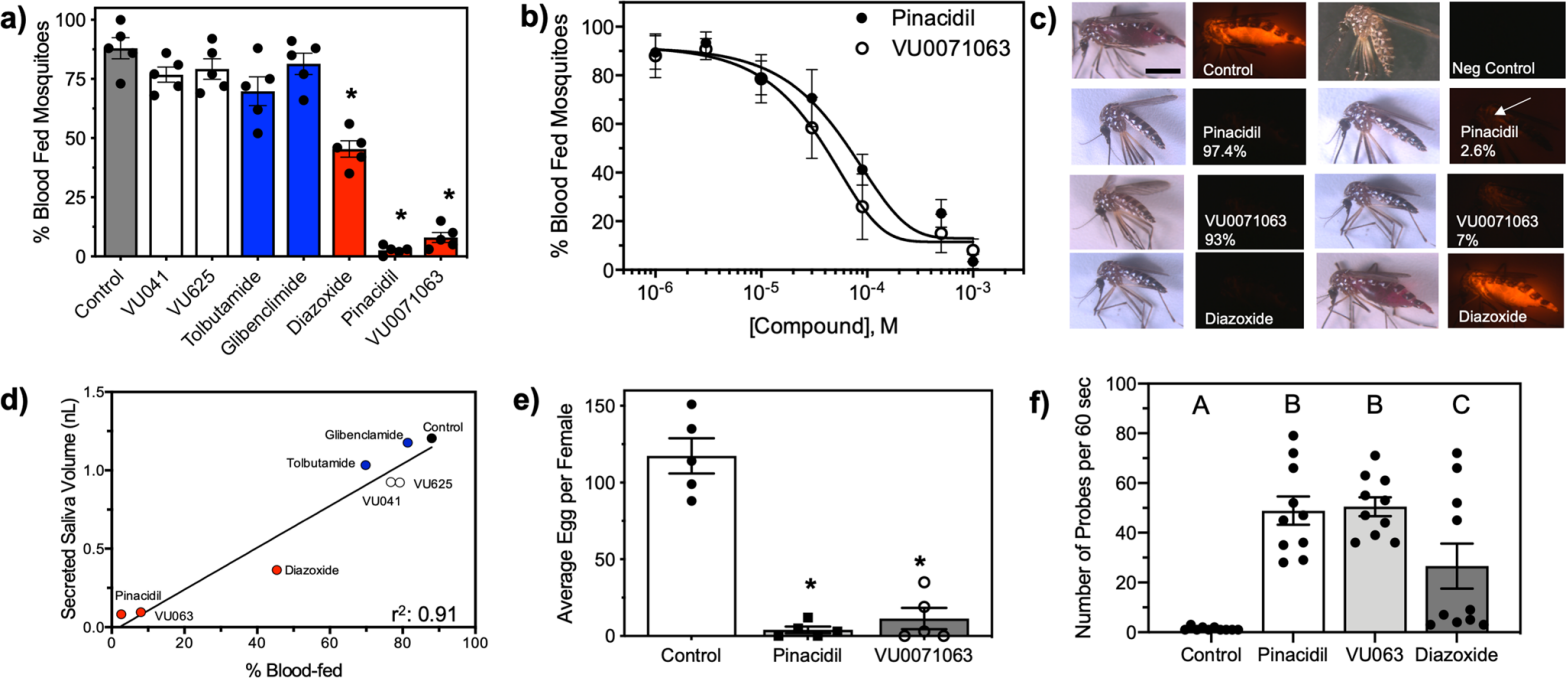
Influence of Kir/K_ATP_ channel modulators to blood-feeding biology of *A. aegypti*. **a** Percentage of mosquitoes that imbibed blood from a membrane feeding system when Kir modulators were dissolved into the blood meal at a concentration of 300–700 µM. Feeding was assessed based on fluorescence resulting from the inclusion of 100 ppm rhodamine B into the blood meal and any fluorescence was considered a fed mosquito. Bars represent mean (*n* = 5) percent feeding with each replicate consisting of 100 mosquitoes and error bars denote SEM. Statistical significance is denoted by the presence of an asterisk with * representing *P* < 0.001. **b** Concentration-response curve for the percentage of blood-fed mosquitoes after exposure to pinacidil (closed circle) or VU0071063 (open circle). Each data point represents mean (*n* = 5) percent fed mosquitoes where each replicate consisted of 30–50 mosquitoes and error bars represent SEM. **c** Representative images of mosquitoes that were provided access to a blood meal treated with RhoB and K_ATP_ activators. Percentages embedded into the image denote the percent mosquitoes that are represented by that image. Scale bar equals 1 mm. **d** linear regression analysis of salivary gland function to blood-feeding efficacy. Goodness-of-fit for the regression is indicated by the *r*^2^ value. **e** Eggs laid per female after feeding on control, pinacidil, or VU0071063 treated blood meals. Bars represent the mean (*n* = 5, 50 individuals per replicate) the number of eggs and error bars represent SEM. Asterisks represent statistical significance at *P* < 0.01 as determined by a one-way ANOVA. **f** Analysis of the number of probes per individual mosquito over a 180 s period. Bars represent mean (*n* = 10) and error bars represent SEM. Bars not labeled by the same letter represent statistical significance at *P* < 0.05. See Supplemental Data 3 for underlying data.

We next aimed to determine if reduced salivary secretions correlated to a reduced ability for blood meal acquisition in mosquitoes by measuring feeding efficiency from *A. aegypti*. The inclusion of K_ATP_ channel activators into the blood meal reduced the imbibed volume of blood and the percent of mosquitoes capable of ingesting blood (Fig. 3a). An average of 88 ± 9% of control mosquitoes were shown to fully engorge themselves on blood, whereas only 2.6 ± 1.3% and 7.1 ± 6% in pinacidil and VU0071063 treatments, respectively imbibed an incomplete blood meal (Fig. 3a). Diazoxide, a third structurally different K_ATP_ activator, reduced the percent of mosquitoes obtaining a blood meal to 45 ± 12% (Fig. 3a). Classical Kir channel modulators (VU041 and VU625) or K_ATP_ channel inhibitors (tolbutamide, glibenclamide) did not alter feeding behavior or the number of mosquitoes capable of imbibing blood (Fig. 3a). Concentration-response curves (CRC’s) were constructed to determine the potency of pinacidil and VU0071063 to inhibit feeding and data show an IC_50_ for VU0071063 to be 38 μM (95% CI: 27–51 μM, Hillslope: 1.5, *r*^2^: 0.93), which was approximately threefold more potent than pinacidil (Fig. 3b). Interestingly, none of the mosquitoes that ingested blood in pinacidil and VU0071063 treatments were capable of fully engorging themselves on the blood meal and of the small percentage that imbibed detectable volumes of blood were found to imbibe significantly less volume than the control (Fig. 3c). Although the total number of mosquitoes that imbibed blood was less in diazoxide treatments, 45% of the mosquitoes that imbibed blood were capable of fully engorging on the blood meal, which was not observed with other K_ATP_ activators (Fig. 3c). Linear regression analysis indicates a tight correlation between reduced blood-feeding and reduced saliva secretions with a goodness of fit (*r*^2^) of 0.91 and a slope of 0.15, which the slope was found to be statistically significant (*P* = 0.0002) deviation from zero (Fig. 3d). The reduced blood ingestion led us to measure the effect on fecundity and pinacidil and VU0071063 significantly (*P* < 0.0001) reduced eggs laid per female when compared to control (Fig. 3e). Control mosquitoes laid 117 ± 29 eggs whereas mosquitoes that fed on pinacidil and VU0071063 treated blood meals laid 4 ± 2 and 11 ± 7 eggs, respectively (Fig. 3e).

### Behavior of mosquitoes feeding on blood treated with K_ATP_ modulators

Video analysis showed interesting shifts in the blood-feeding behavior of mosquitoes feeding on K_ATP_ treated blood versus untreated blood. Mosquitoes that were provided access to K_ATP_ treated blood would probe their mouthparts into the membrane feeder for ~1–2 s, withdraw their mouthparts, move to a new location on the membrane, and initiate probing behavior again (Supplemental Video 1). This repeated probing behavior persisted for ~30 min, but a blood meal was not visibly imbibed during any of these bouts of probing. This was different than behavior observed in control mosquitoes which quickly landed on the membrane, probed, and successfully engorged on the blood meal (Supplemental Video 2). These observations were quantified and mosquitoes provided pinacidil- and VU0071063- treated blood meals probed an average of 50 ± 6 and 51 ± 4 times per minute, respectively which was significantly (*P* < 0.0001) increased from the control that probed an average of 1.4 ± 0.3 times per minute (Fig. 3f). Mosquitoes exposed to diazoxide treated blood showed more variability in their probing than those exposed to pinacidil or VU0071063 with an average of 26 ± 18 probes per minute, which was significantly different from control and pinacidil/VU0071063 (Fig. 3f).

### K_ATP_ Channel activators alter the trafficking of blood meal after ingestion

**Fig. 4:**
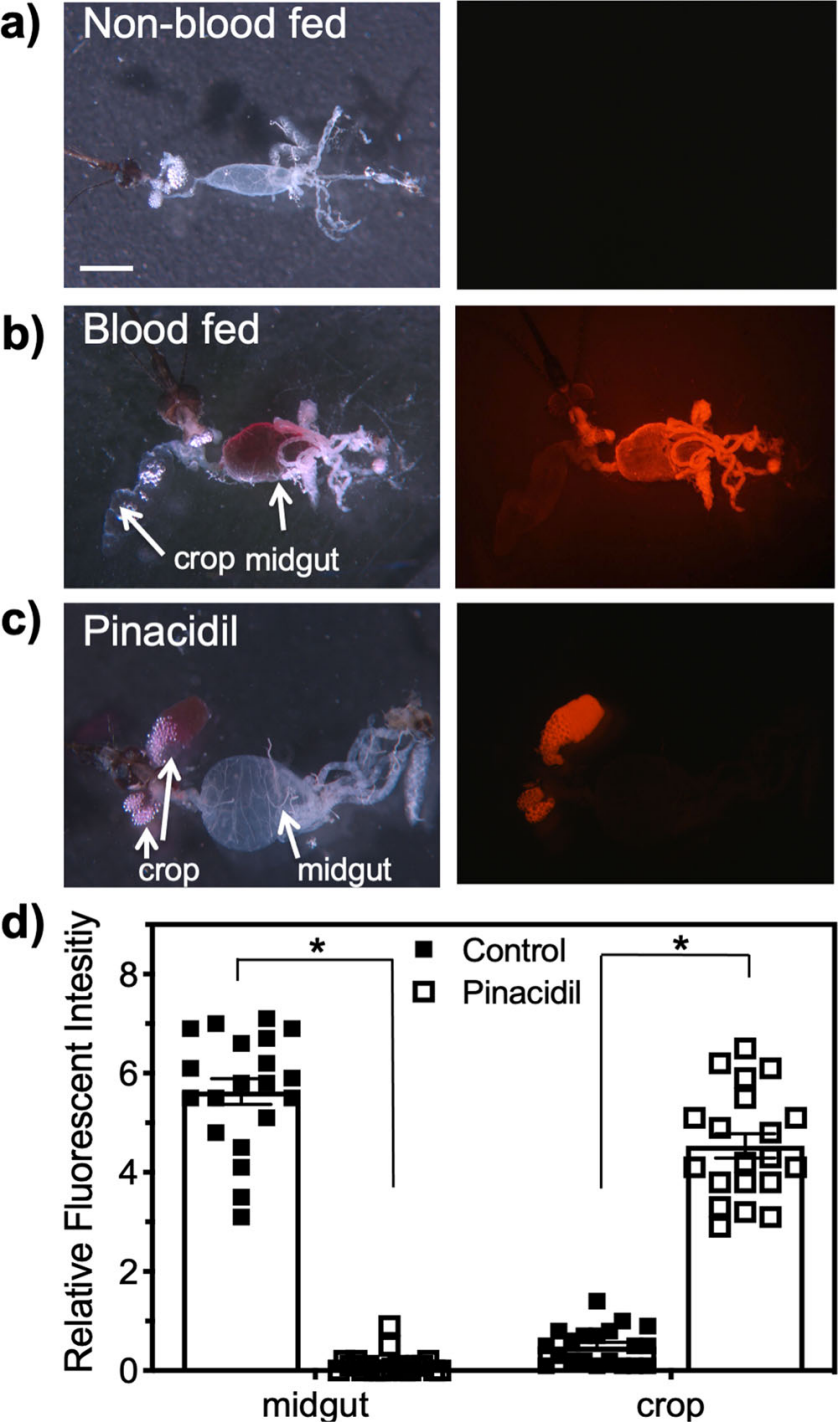
K_ATP_ activators alter the trafficking of blood meal. Representative images of individual mosquitoes indicating altered trafficking to the crop in mosquitoes that were unfed (**a**), blood with solvent only (control, **b**), and fed on pinacidil (**c**) treated blood. Scale bar equals 500 µm. Representative fluorescent images are shown next to white light and were taken by fluorescence microscopy using a rhodamine filter cube (excitation wavelength, 540 nm; emission wavelength, 625 nm) with an exposure time of 300-ms. **d** Blood trafficking to the crop or midgut was quantified by relative Rhodamine B (red) fluorescence intensity. Bars represent mean (*n* = 20 individuals) and error bars represent SEM. Asterisks represent statistical significance at *P* < 0.0001 as determined by a one-way ANOVA with Tukey’s posttest between control and pinacidil treatment groups. See Supplemental Data 4 for underlying data.

Although 97.4% of mosquitoes provided access to pinacidil-treated blood meals did not imbibe any detectable blood, a total of 2.6% of the mosquitoes tested showed a faint and localized rhodamine fluorescent signature (Fig. 3c). We aimed to determine the trafficking pattern of this small volume of blood to assess potential impacts to the reproductive fecundity of vector competency. Control mosquitoes were shown to traffic the blood meal to the mosquito midgut, which is the immediate destination of the blood meal as it is responsible for protein digestion (Fig. 4a, b). Interestingly, pharmacological activation of K_ATP_ channels by pinacidil during blood-feeding caused misdirection of the blood meal as no blood was observed in the midgut of the mosquito and the crop, usually reserved for sugars, was full of blood (Fig. 4c). The fluorophore, Rhodamine B (RhoB), was included in the blood meal to enable relative quantification of blood trafficked to the midgut or crop after exposure to pinacidil. Mosquitoes that fed on control blood meals were trafficked to the midgut with relative fluorescence intensity of 5.5 ± 0.7 whereas the midguts of pinacidil-treated mosquitoes had a relative intensity 25-fold less, which is a statistically significant (*P* < 0.001) reduction. We did not observe any trafficking of the blood meal to the crop in control mosquitoes although misdirection of the blood meal to the crop has been documented to occur in a small proportion of blood-fed mosquitoes^38^. On the contrary, blood was trafficked to the crop in 100% of pinacidil-treated mosquitoes and fluorescence intensity was increased by sevenfold when compared to control mosquitoes, a statistically significant (*P* < 0.001) increase. These data indicate that pinacidil causes misdirection of the blood meal from the midgut to the crop during feeding.

### K_ATP_ channel activators reduce vector competency for Dengue virus 2 (DENV2)

**Fig. 5:**
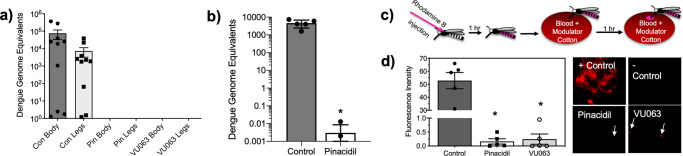
Influence of K_ATP_ activators on the horizontal transmission of DENV2 and a model pathogen from *Aedes aegypti*. **a** DENV2 genome equivalents from bodies and legs of individual mosquitoes 18 days post-feeding on control, pinacidil-, or VU0071063 treated blood meals positive for DENV2. Bars represent mean (*n* = 10, 100 individuals per replicate) and error bars represent SD. **b** DENV2 genome equivalents from blood meals fed on by DENV2-infected mosquitoes. Bars represent mean (*n* = 5) and error bars represent SD. Asterisk represents statistical significance at *P* < 0.0001 as determined by an unpaired *t*-test. **c** Experimental design of horizontal transmission of a model pathogen from *A. aegypti*. **d** Fluorescence units from blood-soaked cotton balls treated with vehicle control, pinacidil (700 µM), or VU0071063 (300 µM) that were fed upon by mosquitoes inoculated with rhodamine B, which was used as a model pathogen. Bars represent mean (*n* = 5) fluorescence units and error bars represent SEM. Note the change in scale of the Y-axis. Asterisks represent statistical significance at *P* < 0.0001 as determined by an unpaired students *t*-test. Representative fluorescent images of control, pinacidil, and VU0071063 treated substrates. White arrows show feeding sites with small levels of fluorescence. See Supplemental Data 5 for underlying data.

The antifeedant and altered trafficking effect of K_ATP_ activators is particularly intriguing from a perspective of vectorial capacity for pathogens because virus acquisition requires the occurrence of blood-feeding events and viral replication in the mosquito relies on the presence of midgut receptors^39^. Thus, we hypothesized that the combination of near elimination of blood-feeding and the altered trafficking of the blood meal to the crop would prevent the acquisition of DENV2. To test this hypothesis, we fed groups of *A. aegypti* on blood meals infected with DENV2 with and without pinacidil or VU063 and measured their ability to acquire and disseminate the virus. Mosquitoes provided access to DENV2 infected blood treated in the absence of K_ATP_ modulators were able to acquire a viral infection and disseminate the virus to the legs within 18 days (Fig. 5a) with an average of 34,286 ± 1449 and 1458 ± 684 DENV2 genome equivalents for the body and legs, respectively (Fig. 5a). These data indicate control mosquitoes were competent vectors at 18 days post-infection. However, mosquitoes exposed to pinacidil or VU0071063 treated blood meals were found to have no detectible amount of DENV2 RNA in the body or legs at 18 days post-infection, indicating treatment with K_ATP_ activators prevented the acquisition of DENV2 (Fig. 5a).

### K_ATP_ channel activators reduce horizontal transmission of a model pathogen by *Aedes aegypti*

The reduction of salivary gland function and feeding after exposure to K_ATP_ channel activators suggested horizontal pathogen transmission is likely to be significantly reduced when specific modulators are included in the feeding substrate. However, current dogma suggests virus-infected saliva is secreted with each probing event and thus, we tested the potential that the increased probing of individual mosquitoes when feeding on K_ATP_ treated blood will increase pathogen transmission. Interestingly, pinacidil-treated blood meals that were fed on by DENV2-infected mosquitoes were shown to have an average of 0.0065 ± 0.002 DENV2 genome equivalents, which is a 686,923-fold reduction of DENV2 titer when compared to control treatment groups (Fig. 5b). The near absence of DENV2 RNA in blood meals provided evidence K_ATP_ agonists prevented horizontal transmission of DENV2 despite the increased probing behavior. We aimed to verify this through the development of an assay enabling visualization of horizontal transmission of a model pathogen with the fluorescent tracer, RhoB (Fig. 5c). RhoB was microinjected into the thorax of female *Ae. aegypti* and allowed to feed on blood-soaked cotton balls that were treated or untreated with K_ATP_ modulators. Control mosquitoes transmitted the model pathogen to the feeding substrate during feeding at an average fluorescence intensity of 49 ± 22 relative units (Fig. 5d), yet mosquitoes that fed on pinacidil or VU0071063 treated blood meals transmitted less model pathogen with average fluorescence intensities of 0.17 ± 0.16 and 0.3 ± 0.25 units, respectively (Fig. 5d), which is a statistically significant reduction (*P* < 0.001). Representative fluorescent images of control, pinacidil, and VU0071063 feeding substrates are shown in Fig. 5d.

### Small-molecule modulators of Kir channels prevents the feeding of *Drosophila melanogaster*

**Fig. 6:**
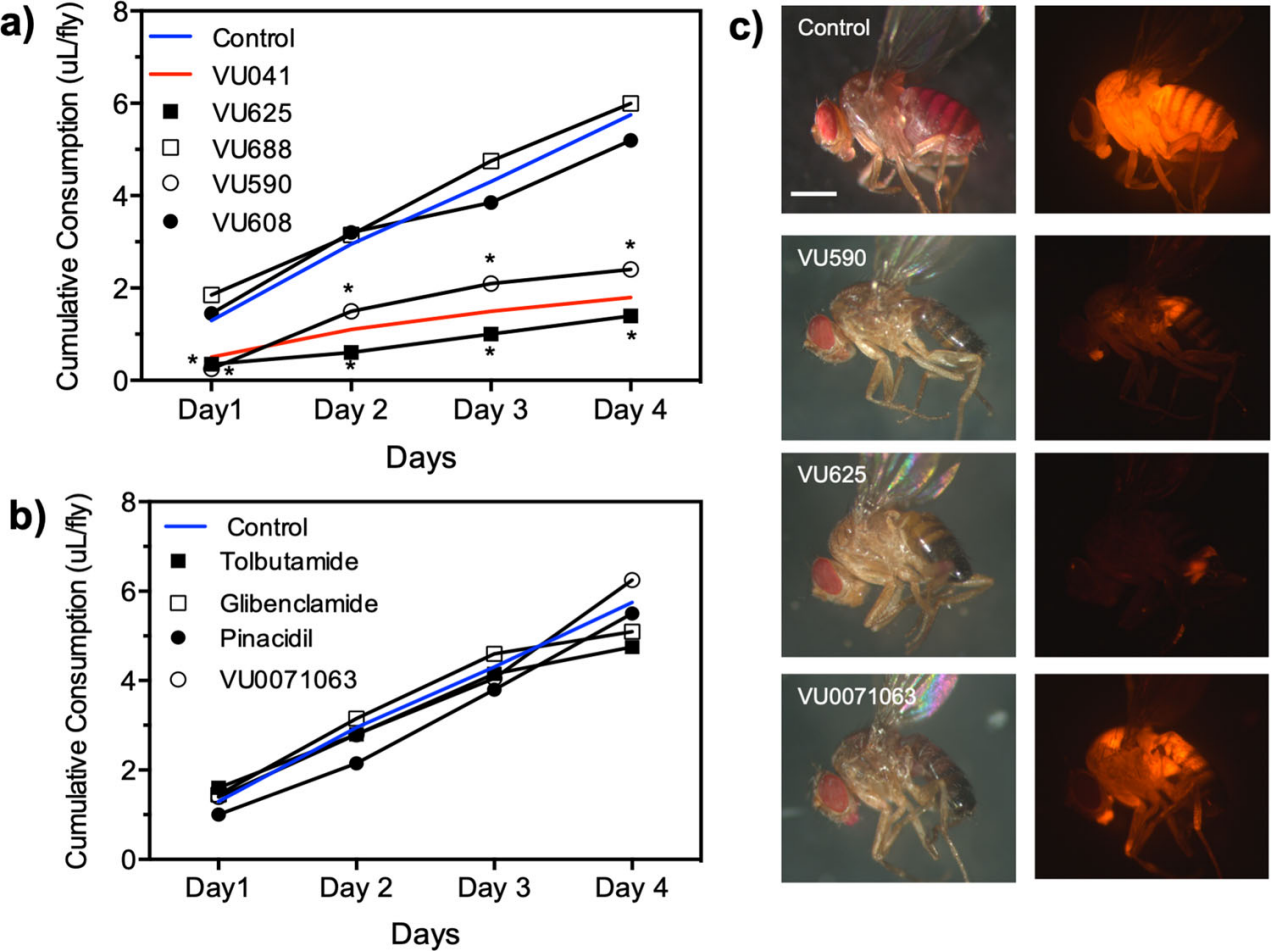
Impact of Kir channel modulators to the non-hematophagous fly, *Drosophila melanogaster*. **a** Measurement of cumulative sucrose consumption by adult *D. melanogaster* over a 4-day period using the CAFE feeding assay with Kir modulators. Total consumption was compared to control flies (blue line) and VU041 (red line). Statistical significance compared to control consumption is denoted by an asterisk where *P < 0.05. **b** Measurement of cumulative sucrose consumption by adult *D. melanogaster* with K_ATP_ channel modulators. For **b**, **c**, data points represent the mean (*n* > 25) cumulative consumption of individual flies. Error bars for panels **b**, **c** have been omitted for clarity. **c** Representative images of reduced feeding are shown by the inclusion of rhodamine B into the sucrose solution and allowing access to feed for 30 min. Scale bar equals 200 µm. See Supplemental Data 6 for underlying data.

To characterize the influence of Kir channels on non-hematophagous fly feeding, we quantified the ingested volume of sucrose after exposure of *Drosophila melanogaster* to pharmacological probes that are structurally distinct and are specific for Kir or K_ATP_ channels (Fig. 6a). Previous work by our group has shown that inclusion of Kir blocker VU041 (200 μM) into a 5% sucrose meal significantly reduced ingestion (Fig. 6a, red line) and salivary gland-specific knockdown of Kir1 reduced feeding efficacy of *Drosophila melanogaster*^33^. Similar to VU041, the classic Kir channel inhibitors VU625 and VU590 significantly (*P* < 0.05) reduced the volume of sucrose ingested at all time points studied when compared to control flies by up to sixfold. Importantly, the ingested volumes of sucrose after flies were exposed to VU688 and VU608, which are the inactive analogs to VU625 and VU590, respectively, were not significantly different when compared to control or active analog treatments (Fig. 6a).

To decipher whether classic or ATP-gated Kirs are responsible for sucrose feeding in *Drosophila*, we employed two activators (pinacidil, VU0071063) and two inhibitors (tolbutamide, glibenclamide) of K_ATP_ channels as probes to measure their influence on sucrose consumption. Contrary to classic Kir channel inhibitors, no K_ATP_ channel modulator altered sucrose consumption of *Drosophila* (Fig. 6b). Representative images of flies that fed on Kir or K_ATP_ modulators are shown in Fig. 6c.

## Discussion

The salivary glands of mosquitoes exhibit two characteristics that make them compelling objects of study. First, these glands facilitate blood-feeding through salivary secretions that carry anticoagulants, immunomodulatory factors, and vasodilators^36,40,41^ and second, acquisition and horizontal transmission of pathogens rely on the proper function of the salivary gland as both rely on active feeding and salivation. It is logical to suggest that elimination of saliva would prevent mosquito blood-feeding and disease pathogenesis^42^, yet seminal work reduced enthusiasm for targeting mosquito salivary gland function as a mechanism for antifeedant development based on evidence that surgical deprivation of saliva through transection of the salivary duct did not inhibit the ingestion of blood^26,43^. Due to this, significant efforts have been put forth to understand the salivary constituents that are essential for blood-feeding events and contribute to horizontal transmission of pathogens to guide the development of vaccines that impede blood-feeding^17,44^ or pathogen transmission^14,45,46^. Molecular and genetic analyses of mosquito feeding have led to significant advancements in mosquito feeding biology and siaolomics, while indirectly generating significant knowledge gaps pertaining to the fundamental underpinnings enabling the function of the mosquito salivary gland. Our data clearly indicate that transection of the mosquito salivary duct inhibits the secretion of saliva after stimulation with dopamine (Fig. 1), which has been shown to be a stimulant for mosquito salivation^47^, and lack of salivation was strongly correlated to an inability to blood feed (Fig. 1). These data challenge the current paradigm that indicates mosquito salivation is not a prerequisite to blood-feeding by providing evidence that blood-feeding is likely dependent on salivation as has been documented in other arthropods^30–32^. Thus, this study was driven by the premise that attenuation of blood-feeding behavior through inhibition of salivary gland function will reduce horizontal transmission and the burden of disease.

Original work indicated surgical deprivation of saliva through transection of the salivary duct inhibited allergic reaction in the human host, but transection did not inhibit the ingestion of blood and trafficking of the blood to the midgut^26,43^. These data led to the conclusion that, contrary to other arthropod species^29,48^, the primary role of mosquito saliva is for vessel localization and is not a prerequisite to blood-feeding^24–26,49^. These data are opposite to those reported in this study and maybe because these experimental mosquitoes were not completely deprived of saliva, which is unknown because quantification of secreted saliva from individuals that were shown to feed was not performed and prevented verification of duct transection. Thus, it is possible, albeit speculative, these experimental mosquitoes had partial transections of the salivary duct that reduced the concentration of saliva proteins below the threshold for eliciting an immune reaction in the host and not impeding blood meal ingestion. Accordingly, we manually transected the salivary duct and measured fluid secretion rates and blood-feeding ability on artificial membrane systems. A total of 13% of the mosquitos were able to imbibe a complete blood meal after transection of the salivary duct whereas the remainder of the mosquitoes did not imbibe blood, which mirrors previous reports^24–26^. We subsequently quantified saliva secretion from the mosquitoes that were able to feed to measure fluid secretion and verify the salivary duct was indeed transected. Importantly, all the mosquitoes that imbibed blood secreted approximately half the salivary volume of intact controls, which suggests transection of the duct was incomplete or unsuccessful. These data indicate previous studies may have measured blood-feeding ability in mosquitoes that were capable of secreting saliva due to a partial transection or rejoining of the ducts but were under the assumption these mosquitoes were saliva deprived. Considering this, the data shown in Fig. 1 indicate saliva is indeed a prerequisite to mosquito blood-feeding, and therefore, the mosquito salivary gland represents a target tissue for the development of mosquito antifeedants.

While studies have shown the secretory activity of mosquito salivary glands is regulated by dopamine, serotonin, and pilocarpine^37,47^, the regulatory role of ion channels in the secretory activity of the mosquito salivary gland has remained undetermined. Kir channels have been shown to constitute a critical conductance pathway in mosquito Malpighian tubules^50–52^, which are similar to salivary glands from a physiological perspective as both are polarized epithelial tissue responsible for fluid secretion. *A. aegypti* tubules express Kir1, Kir2, and Kir3 mRNAs where they contribute to transepithelial fluid and K^+^ secretion that raised the potential Kir channels are expressed and physiologically relevant in *A. aegypti* salivary glands. Indeed, isolated salivary glands were found to abundantly express Kir2A but no expression of Kir1, which is the Kir encoding gene expressed in *D. melanogaster* salivary glands^27,33,53,54^. The secretory activity of the mosquito salivary gland was found to be sensitive to K_ATP_ channel agonists (pinacidil, VU0071063) that were also shown to nearly eliminate blood ingestion. Classical Kir channel modulators (VU041, VU625) or K_ATP_ channel antagonists (tolbutamide, glibenclamide) did not alter fluid secretion or blood ingestion, which was similar to that observed with the Lonestar tick, *Amblyomma americanum*^29^. Interestingly, sucrose ingestion by *D*. melanogaster, a non-hematophagous dipteran, was not significantly altered by K_ATP_ channel activators or inhibitors but significantly reduced by VU041 and VU625, two Kir1 inhibitors, which was similar to that observed with the non-hematophagous cotton aphid, *A. gossypii*. These data suggest hematophagous arthropods, such as ticks and mosquitoes, have evolved the use of K_ATP_ channels to regulate the secretory activity of the salivary gland and blood-feeding over classical Kir channels due to the phagostimulant activity of ATP in hematophagous arthropods but not sap-feeding insects^55^.

Vectorial competency is generally defined as the ability for a given vector to acquire and subsequently transmit a pathogen, which requires the ability to blood feed. For mosquito arboviruses, the virus is acquired by an adult female mosquito during blood-feeding from a viremic host and subsequently undergoes replication in midgut epithelial cells. Failure of the virus to reach the midgut cells will prevent virus replication and thus, reduce or eliminate vector competency. After virus replication, the virus migrates to the salivary gland and is horizontally transmitted during blood-feeding to a new host and thus, saliva secretion has significant implications for the manifestation of disease in vertebrates because the volume of saliva secreted into the host is directly correlated to pathogen transmission and disease severity^42,56^. Taken together, K_ATP_ agonists are likely to inhibit vector competency through reduced DENV2 acquisition due to a reduced blood meal size (Fig. 3) and altered trafficking of the blood meal away from the midgut epithelium (Fig. 4), as well as reduced horizontal transmission due to reduced feeding efficacy (Fig. 3). Indeed, mosquitoes provided access to DENV2-infected blood meals treated with pinacidil or VU0071063 resulted in zero detectable DENV2 RNA in the whole body or legs of individual mosquitoes. While these data are not surprising considering the lack of blood acquisition in most mosquitoes and altered trafficking of the blood to the crop, the lack of horizontal transmission after exposure to K_ATP_ agonists was surprising considering the increased probing events observed in K_ATP_ agonist treatment groups (Fig. 3f). Current dogma indicates saliva and arboviruses^57^ are secreted during probing events, suggesting that K_ATP_ agonists would increase horizontal transmission of arboviruses due to the dramatic increase in probing compared to controls (Fig. 3f). However, pinacidil-treated blood meals had very low levels of DENV2 after feeding whereas control blood meals contained a high titer of DENV2. To verify these data, we developed a model transmission assay with RhoB (Fig. 4c) that allowed for visualization and quantification of model pathogens horizontally transmitted during *A. aegypti* feeding. These data support the qPCR data that indicated no horizontal transmission of DENV2 by clearly showing pinacidil and VU0071063 nearly eliminated the horizontal transmission of the model pathogen to blood-soaked cotton balls. The lack of correlation between probe events and horizontal transmission of DENV2 and the model pathogen after exposure to K_ATP_ agonists suggests that inhibition of the secretory activity of the salivary gland precludes horizontal transmission of pathogens despite secretion of saliva during probing. Although further analysis is warranted, it is possible the saliva secreted during probing is not derived from the salivary gland, which contains the pathogen, but is a saliva-like fluid stemming from fluid transport across the walls of the salivary ducts^26^. To this point, we suspect this non-salivary gland derived fluid was what was measured in mosquitoes with transected salivary ducts that were shown to secrete small quantities of saliva but were unable to imbibe blood (Fig. 1e).

The data presented in this study provide evidence that *A. aegypti* salivary gland function is indeed a prerequisite for successful blood-feeding that supports the notion the mosquito salivary gland represents a target tissue for the development of antifeedant and anti-transmission technologies in mosquitoes. Further, these data provide evidence that although low volumes of saliva are secreted after exposure to K_ATP_ agonists or transection of the mosquito duct, horizontal transmission of pathogens does not occur despite increased probing events. The presented data provide the first evidence that K_ATP_ channels are critical for mosquito blood-feeding events and vector competency in mosquitoes, which has remained understudied and expands previous studies that analyzed the role of neurochemical mechanisms in saliva secretion^37,47^. Taken together, the data presented in this study indicate mosquito salivary gland function is tied to blood-feeding behavior and provide evidence that salivary gland-specific K_ATP_ channels represent putative therapeutic target sites to reduce the health and economic burden stemming from mosquito hemaztophagy.

## Methods

### Pharmacological modulators

Kir channel modulators VU041 and VU625 were originally identified in high-throughput screens to identify inhibitors of mosquito Kir1 channels^58,59^ and were purchased in bulk by custom synthesis from Molport Inc. (Riga, Latvia). K_ATP_ modulators pinacidil, tolbutamide, diazoxide, and glibenclamide were purchased from Sigma-Aldrich (St. Louis, MO, USA) and VU0071063 was purchased by custom synthesis from Molport Inc. All chemical modulators used in this study were verified by Molport or Sigma-Aldrich to be >95% pure.

### Arthropod and DENV2 culture

*A. aegypti* Paea strain was used for this study that was originally collected in French Polynesia in 1993^60^ and was generously provided by Research Infrastructures for the Control of Vector-Borne Diseases (Infravec2). This mosquito strain was used because it is highly susceptible to DENV2 infections and is routinely used as a control in vector competence experiments^60^. Mosquitoes used were 3–7 days old and reared in an environmental chamber set to 27 °C and 75% humidity in the Life Sciences Building of Louisiana State University (Baton Rouge, LA, USA)^47^. DENV type 2 strain 1232 was used in this study and was donated by Dr. Rebecca Christopherson (Louisiana State University, Department of Pathobiological Sciences) and was originally isolated from a human patient in Jakarta, Indonesia in 1978. This strain of DENV2 was selected for this study because needle inoculation and oral infection has been shown to result in 100% infection^61^. DENV2 was inoculated on confluent Vero cells grown at 37 °C and 5% CO_2_ in Medium-199 with Earl’s salts, supplemented with penicillin/streptomycin/Amphotericin B and 10% FBS. The supernatant was harvested 5 days post-inoculation, titrated by plaque assay and qRT-PCR, and used at a concentration of 3e7 plaque-forming units per mL^62^.

### Transection of the salivary duct

**Fig. 7:**
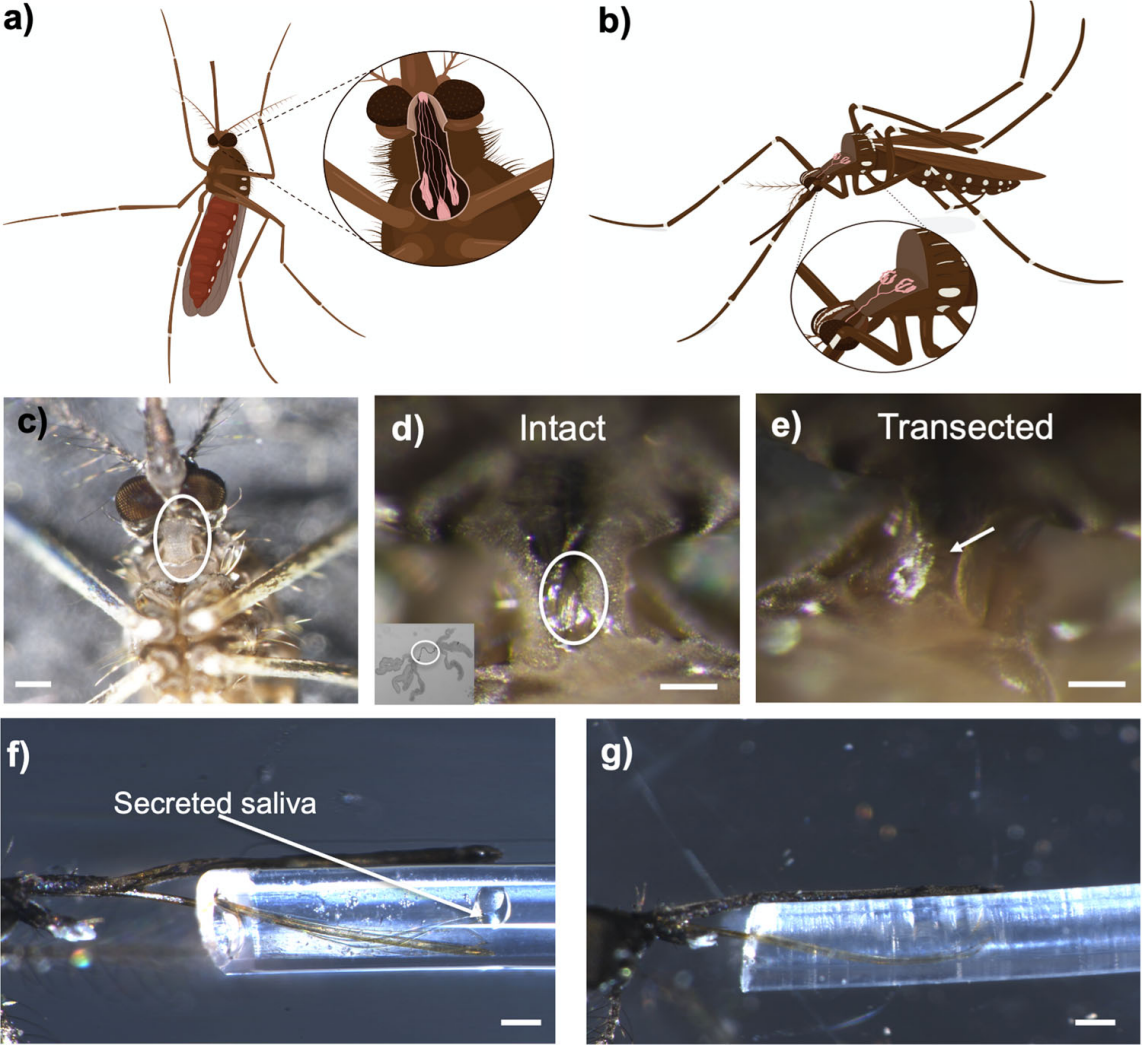
Methodological overview of salivary duct transection and verification of transection through modified Ramsay assay. Drawing of ventral (**a**) and dorsal (**b**) view of mosquito body showing the salivary ducts and salivary glands. The ventral view shows the proventriculus and ingestion ducts. The ventral side of female *A. aegypti* clearly shows the neck membrane (white circle) which encases the salivary duct. Drawings were made by the Ella Maru Studio and were released to Dr. Daniel Swale (Department of Entomology, Louisiana State University). **c** Ventral view of *A. aegypti* with white circle highlighting the neck membrane that encases the salivary ducts. The neck membrane was not removed prior to transection of the duct to reduce negative consequences to the mosquito and increase the rate of survival. Scale bar equals 200 µm. **d**, **e** Representative images of intact (**d**) and transected (**e**) salivary duct after the removal of the neck membrane. The white circle on panel **d** shows the intact salivary duct with the inset image showing the dissected salivary glands with the white circle showing the ducts. The white arrow on panel **d** indicates the lack of salivary ducts post-transection. The scale bar on panels **d**, **e** equals 75 µm. **f**, **g** Representative images of fluid secretion from a mosquito with an intact (**f**) or transected (**g**) salivary duct. Scale bars on panels **f**, **g** equals 300 µm.

Transection of the female mosquito salivary duct was modified from the original methodological description^26^ and is shown in Supplemental Video 3 and drawings of ventral and dorsal mosquito highlighting the location of the salivary gland and duct location are shown in Fig. 7a, b, respectively. We first performed salivary duct transections by removing the neck membrane (Fig. 7c, white circle) and cutting the duct with minutien insect pins. However, these mosquitoes displayed a high mortality rate due to the invasiveness of the technique. Thus, we used these dissections to familiarize ourselves with the specific location of the salivary duct that allowed subsequent dissections to be minimally invasive and increased the likelihood of survival after the microsurgery. Female mosquitoes were anesthetized on ice, transferred to a wax dish, and oriented ventral side up to expose the mosquito neck membrane (Fig. 7c, white circle). Using a Nikon SMZ1270i dissection scope, a small incision on the neck membrane was made where the common salivary duct was predetermined to be located using Vannas Spring Scissors with a 2 mm cutting edge (Fine Science Tools, Foster City, CA, USA). Representative images of the intact and transected salivary duct with the neck membrane removed are shown in Fig. 7d, e, respectively. Transected mosquitoes were maintained at 27 °C for 60 min to ensure survival and normal ambulatory and flight behavior. Mosquitoes not able to fly 60-min post-surgery were discarded from the study. Transected mosquitoes were later used for salivation assays or blood-feeding. For the blood-feeding assays, mosquitoes were starved overnight before the transection to increase blood-feeding success rates. Saliva secretion was quantified using a modified Ramsay assay^47^ immediately after blood-feeding from all transected mosquitoes to ensure transection of the salivary duct was complete in the individuals capable of imbibing blood. Representative images of salivation from intact and salivary gland transected mosquitoes are shown in Fig. 7f, g, respectively.

### Saliva quantification

Secreted saliva was quantified followed previously described methods that are redescribed below^47^. Mosquito appendages were removed to prevent mobility. The mosquito stylet sheath was disassociated with forceps and stylets were inserted into PE tubing (0.5 mm OD, 0.25 mm ID) filled with heavy mineral oil. To quantify saliva secretion after salivary duct transection, individual mosquitoes were intrathoracically injected with 69 nl of 100 µM dopamine (DA) HCl dissolved with PBS via World Precision Instruments (WPI) Nanoliter2010 nanoliter injector operated by a WPI SMARTouch controller and the diameter of the saliva droplet was measured 10-min post DA injection with Nikon SMZ1270i stereomicroscope equipped with a DS-Ri2 camera and Nikon Elements Professional software. The total volume of secreted saliva was quantified with the formula V = 4/3πr^3^ ^47^.

To test the role of Kir channels to salivary gland function, Kir channel modulators were solubilized in DMSO and subsequently dissolved in PBS with added calcium. Kir channel modulators were injected (69 nl) at a discriminatory concentration of 500 µM. Control groups were injected with vehicle dissolved in PBS. Mean secretion rates were compared through one-way ANOVA with Tukey’s posttest. Concentration-response curves were constructed to determine the potency of pinacidil and VU0071063 where the curve consisted of 6–8 concentrations and each concentration contained an *n* = 5 replicates where each replicate consisted of 25 mosquitoes. IC_50_ values for pinacidil- and VU063-mediated salivary and blood-feeding inhibition were obtained through variable slope nonlinear regression using a Hill equation in GraphPad Prism^TM^ (GraphPad Software, San Diego, CA, USA).

### Kir channel expression

qRT-PCR was used to assess the expression of *A. aegypti* Kir channel subunits in the adult female salivary glands^34,51^. The salivary gland was dissected from ten individual female *A. aegypti* adults and total RNA was isolated using Trizol reagent and PureLink RNA Mini Kit (Thermo Fisher Scientific, USA) was used according to manufacturer instructions. Purified RNA was treated with DNase I (amplification grade, Thermo Fisher Scientific). cDNA was synthesized using SuperScript III First-Strand Synthesis System with Oligo(dT)_20_. Primer pairs for *Ae*Kir channels and *Ae*Kir2A splice variants were used as described in previous work^34,51^. The housekeeping gene RPS7 was used as an internal positive control as well as a loading control to ensure that each sample was loaded equally on each lane of the gel.

### Quantification of blood meal trafficking

The fluorophore, rhodamine B (100 ppm), was included in the blood to enable relative quantification of ingested blood^30,33,63^. To measure Rhodamine B fluorescence, which was used as a proxy for blood ingestion, digital images were captured with AxioVision version 4.6 (Carl Zeiss) and were observed under fluorescence microscopy using a rhodamine filter cube (excitation wavelength, 540 nm; emission wavelength, 625 nm). All fluorescent images were captured at an exposure time of 300-ms. Minimal to no auto-fluorescence of the negative control negated the need to optimize the fluorescence exposure time. To perform relative quantification of ingested blood, we analyzed the fluorescent images with ImageJ (NIH, Bethesda, MD, USA)^64^ by selecting the midgut or crop region of the mosquito using the selection tool and subsequently measuring the intensity of red wavelengths in the selected area through the RGB Measure plugin available in the software. Relative fluorescent units were determined by mean intensity/area. The mean (*n* = 5 replicates, with ten individuals in each replicate) fluorescence intensity for the pinacidil treatment group was determined and statistically compared to control by an unpaired *t*-test for the crop and midgut.

### DENV2 RNA extraction and quantitative-PCR

DENV2 RNA was extracted from the body (head, thorax, and abdomen) and leg homogenates with MagMAX™-96 Viral RNA Isolation kit (Applied Biosystems, AM1836) as per manufactures instructions. Blood samples were processed with MagMAX™ Viral/Pathogen kit (Applied Biosystems, A42352) according to the manufacturer’s instruction. The purification procedure was conducted with KingFisher Duo Prime nucleic acid purification platform and extracted RNA was used for viral quantification with qRT-PCR with SuperScript™ III Platinum™ One-Step qRT-PCR kit (Thermo Fisher Scientific, Waltham, MA, USA). qRT-PCR singleplex assay was performed using specific primers and probes for DENV2^65^. Primers DENV2 forward primer (5′-CAGGTTATGGCACTGCGAT-3′), reverse primer (5′-CCATCTGCAGCAACACCATCTC-3′), and probe (5′-/56-FAM/CTCTCCGAG/ZEN/AACAGGCCTCGACTTCAA/3IABkFQ/-3′) were synthesized by Integrated DNA Technologies^®^ Inc and DENV was quantified in mosquito tissues or blood using QuantStudio 6 Flex Real-Time qPCR System (Applied Biosystems™).

### DENV2 acquisition assay

Defibrinated rabbit blood was infected with 3e5 pfu/mL of DENV2 and 100 naïve, 4-day-old female *A. aegypti* were provided access to the infected blood meal through the Hemotek (Blackburn, UK) artificial feeding apparatus. Blood-fed females were selected through Rhodamine fluorescence and transferred into holding chambers and were kept at 27 °C and 80% RH to facilitate viral propagation. Mosquito legs and abdomens were dissected at 18 days post blood-feeding and subjected to viral quantification through qRT-PCR. Experiments were reproduced five separate times with 30–50 individuals used for each replicate.

### DENV2 horizontal transmission assay

Six-day-old adult female mosquitoes were infected through needle inoculation with 10^7^ pfu/mL of DENV2 and maintained for 7 days. Groups of 100 females were allowed to probe and feed on 0.2 mL of defibrinated rabbit blood (Hemostat Laboratories, Dixon, CA) using the Hemotek system with the 0.3 mL feeding chambers. The mosquitoes were provided access to the blood meal for 90 min. After 90 min, the blood was collected and immediately subjected to viral RNA extraction and qPCR. Experiments were repeated on three separate cohorts of mosquitoes.

### Horizontal transmission of a model pathogen

Female *A. aegypti* aged 4–5 days were deprived of sucrose for 12 h, cold anesthetized to reduce movement, and microinjected with 69.0 nL of 200 µM Rhodamine B (RhoB) solution. Mosquitoes were maintained at 28 °C/75% RH for a 60-min and subsequently transferred into 50 mL plastic conical vials with a cotton ball soaked with defibrinated bovine blood. Pinacidil-treated blood was prepared by initially dissolving pinacidil or VU0071063 in DMSO and diluting it into the blood to a final concentration of 2 mM (DMSO 0.5%, v/v). Vehicle control groups fed on blood treated with DMSO at 1% v/v. Mosquitoes were removed from the conical tubes after 2 h, and fluorescent images of cotton balls were captured with AxioVision version 4.6 (Carl Zeiss) under fluorescence microscopy using a rhodamine filter cube (excitation wavelength, 540 nm; emission wavelength, 625 nm). All fluorescent images were captured at an exposure time of 300-ms. Minimal to no auto-fluorescence of the negative control negated the need to optimize the fluorescence exposure time. To perform relative quantification of horizontal transmission of RhoB, we analyzed the fluorescent images with ImageJ (NIH, Bethesda, MD, USA) by selecting the cotton ball using the selection tool and subsequently measuring the intensity of red wavelengths in the selected area through the RGB Measure plugin available in the software. Relative fluorescent units were determined by mean intensity/area. The mean (*n* = 5 replicates, where each replicate consisted of ten individuals) fluorescence intensity for each treatment group was determined and statistically compared with a one-way ANOVA with multiple comparisons posttest.

### Statistics and reproducibility

Statistical analyses were performed using GraphPad Prism^TM^ (GraphPad Software, San Diego, CA, USA). Details regarding the statistical tests and definitions of significance are provided in the corresponding methods section for each analyses and/or the figure legends. Data were reported as mean ± SEM unless specified otherwise where the means were collected from a minimum of three replicates where each replicate consisted of at least ten individuals. Specifics on the number of replicates and individuals used for each experiment are included in the corresponding methods section and/or figure legends.

### Reporting summary

Further information on research design is available in the Nature Research Reporting Summary linked to this article.

## Supplementary information

Supplementary Information

Description of Additional Supplementary Files

Supplemental Video 1

Supplemental Video 2

Supplemental Video 3

Supplemental Data 1-6

Reporting Summary

## Data Availability

The data depicted by the figures are contained within Supplementary Data 1–6. The datasets generated during and/or analyzed during the current study are also available from the corresponding author on reasonable request.
