# Supplementary material for: ATP-sensitive inward rectifier potassium channels reveal functional linkage between salivary gland function and blood feeding in the mosquito, Aedes aegypti
Source: Commun Biol. 2022 Mar 28;5:278. doi: 10.1038/s42003-022-03222-1 (PMC8960802; doi:10.1038/s42003-022-03222-1)
Supplement: Supplementary file 1 — Supplementary Information [file 42003_2022_3222_MOESM1_ESM.pdf]

Supplemental Information

Title: ATP-sensitive inward rectifier potassium channels reveal functional linkage between salivary gland function and blood feeding in the mosquito, *Aedes aegypti*

Short Title: Novel mechanism to prevent mosquito feeding

Authors: Zhilin Li[1], Alexander Hoosoo-Hui[1], Flinn O'Hara[1], Daniel R. Swale[1*]

Author Affiliations:

[1] Louisiana State University AgCenter, Department of Entomology, Baton Rouge, LA 70803, USA

Video Legends

Supplemental Video 1. Feeding behavior of *A. aegypti* during blood feeding on pinacidil treated blood meal. 100 female *A .aegypti* mosquitoes were provided access to a pinacidil treated blood meal through Hemotek feeding system. Mosquitoes were observed to probe the blood meal for one to three seconds, remove their mouthparts, and reattempt feeding. Video was recorded during the 60-minute feeding period using a Nikon D750 camera with a AF-S DX Micro-NIKKOR 40 mm f/2.8G lens.

Supplemental Video 2. Feeding behavior of *A. aegypti* during blood feeding on blood treated with vehicle (DMSO) only. 100 female *A .aegypti* mosquitoes were provided access to a blood meal through Hemotek feeding system. Mosquitoes were observed to land on the blood meal, insert their mouthparts, and begin engorging on blood. Video was recorded during the 60-minute feeding period using a Nikon D750 camera with a AF-S DX Micro-NIKKOR 40 mm f/2.8G lens.

Supplemental Video 3. Transection of the salivary duct. Video showing methods of transecting the salivary duct with a minimally invasive approach. All mosquitoes were tested for ability to salivate through the Ramsay assay to verify transection of the salivary duct after microdissection surgery.

Supplemental Figures

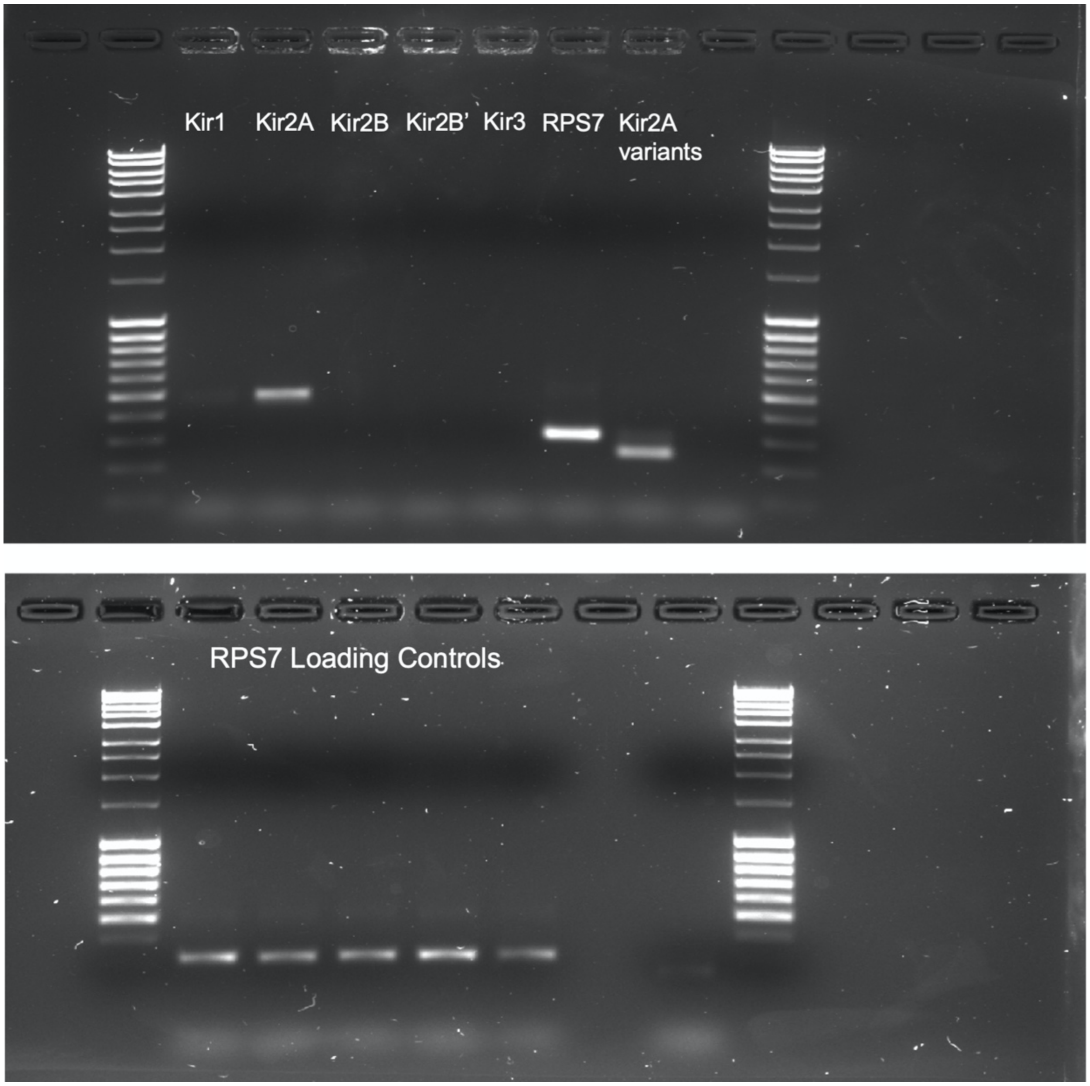

Supplemental Figure 1. Expression of genes encoding Kir channel subunits in the adult female *A. aegypti* salivary gland and corresponding RPS7 loading controls. This is an uncropped version of the gel shown in Figure 2A.

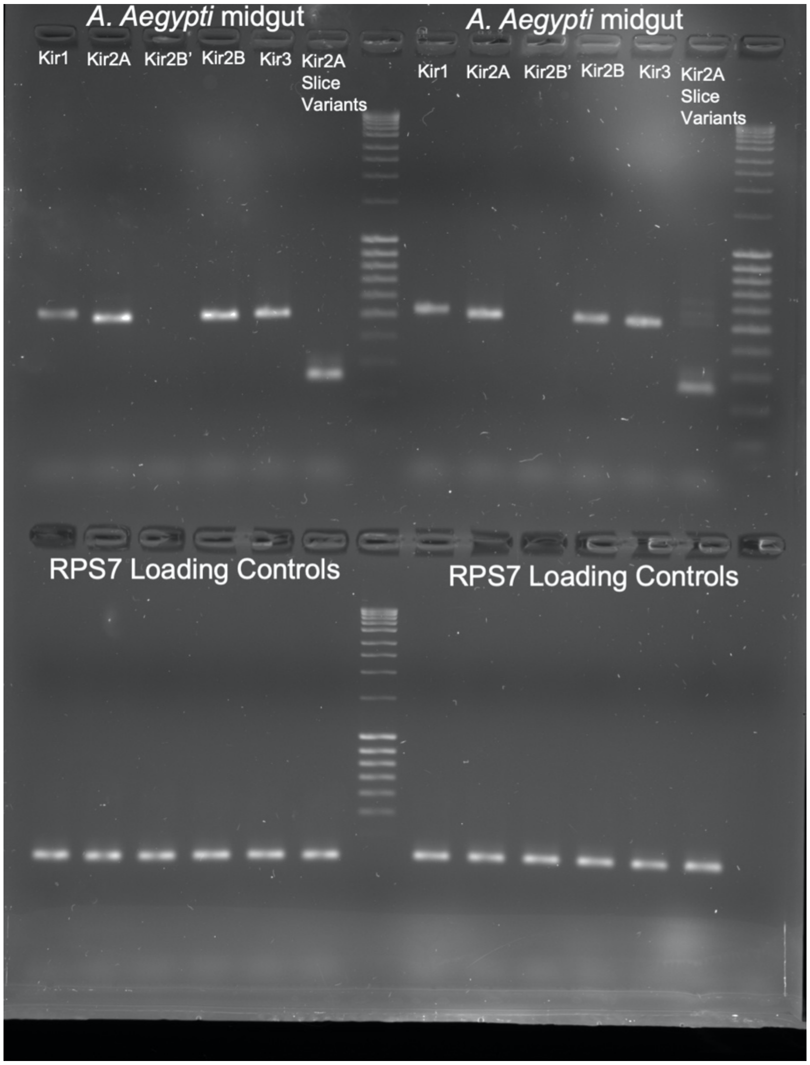

Supplemental Figure 2. Expression of genes encoding Kir channel subunits in the adult female *A. aegypti* midgut. The results of RT-PCR reaction are shown after simultaneous amplification of specific Kir channel genes and a corresponding control gene (RPS7). This reaction was performed in triplicate and the image shows a representative image. Asterisk represents 1000 bp on the Thermo Scientific MassRuler DNA Ladder.
