# Supplementary material for: ATP-sensitive inward rectifier potassium channels reveal functional linkage between salivary gland function and blood feeding in the mosquito, Aedes aegypti
Source: Commun Biol. 2022 Mar 28;5:278. doi: 10.1038/s42003-022-03222-1 (PMC8960802; doi:10.1038/s42003-022-03222-1)
Supplement: Supplementary file 2 — Description of Additional Supplementary Files [file 42003_2022_3222_MOESM2_ESM.pdf]

Description of Additional Supplementary Files

File name: Supplemental Video 1.
Description: Feeding behavior of A. aegypti during blood feeding on pinacidil treated blood meal. 100 female A .aegypti mosquitoes were provided access to a pinacidil treated blood meal through Hemotek feeding system. Mosquitoes were observed to probe the blood meal for one to three seconds, remove their mouthparts, and reattempt feeding. Video was recorded during the 60-minute feeding period using a Nikon D750 camera with a AF-S DX Micro-NIKKOR 40 mm f/2.8G lens.

File name: Supplemental Video 2.
Description: Feeding behavior of A. aegypti during blood feeding on blood treated with vehicle (DMSO) only. 100 female A .aegypti mosquitoes were provided access to a blood meal through Hemotek feeding system. Mosquitoes were observed to land on the blood meal, insert their mouthparts, and begin engorging on blood. Video was recorded during the 60- minute feeding period using a Nikon D750 camera with a AF-S DX Micro-NIKKOR 40 mm f/2.8G lens.

File name: Supplemental Video 3.
Description: Transection of the salivary duct. Video showing methods of transecting the salivary duct with a minimally invasive approach. All mosquitoes were tested for ability to salivate through the Ramsay assay to verify transection of the salivary duct after microdissection surgery.

File name: Supplemental Data 1-6
Description: Data depicted by the figures.
