# Supplementary material for: ATP-sensitive inward rectifier potassium channels reveal functional linkage between salivary gland function and blood feeding in the mosquito, Aedes aegypti
Source: Commun Biol. 2022 Mar 28;5:278. doi: 10.1038/s42003-022-03222-1 (PMC8960802; doi:10.1038/s42003-022-03222-1)
Supplement: Supplementary file 7 — Reporting Summary [file 42003_2022_3222_MOESM7_ESM.pdf]

nature research

Corresponding author(s): Daniel R Swale
Last updated by author(s): Dec 14, 2021

Reporting Summary

Nature Research wishes to improve the reproducibility of the work that we publish. This form provides structure for consistency and transparency in reporting. For further information on Nature Research policies, see our Editorial Policies and the Editorial Policy Checklist.

Statistics

For all statistical analyses, confirm that the following items are present in the figure legend, table legend, main text, or Methods section.

n/a	Confirmed	
☐	☒	The exact sample size (*n*) for each experimental group/condition, given as a discrete number and unit of measurement
☐	☒	A statement on whether measurements were taken from distinct samples or whether the same sample was measured repeatedly
☐	☒	The statistical test(s) used AND whether they are one- or two-sided<br>*Only common tests should be described solely by name; describe more complex techniques in the Methods section.*
☐	☒	A description of all covariates tested
☐	☒	A description of any assumptions or corrections, such as tests of normality and adjustment for multiple comparisons
☐	☒	A full description of the statistical parameters including central tendency (e.g. means) or other basic estimates (e.g. regression coefficient) AND variation (e.g. standard deviation) or associated estimates of uncertainty (e.g. confidence intervals)
☐	☒	For null hypothesis testing, the test statistic (e.g. F, t, r) with confidence intervals, effect sizes, degrees of freedom and P value noted<br>*Give P values as exact values whenever suitable.*
☒	☐	For Bayesian analysis, information on the choice of priors and Markov chain Monte Carlo settings
☒	☐	For hierarchical and complex designs, identification of the appropriate level for tests and full reporting of outcomes
☐	☒	Estimates of effect sizes (e.g. Cohen's d, Pearson's r), indicating how they were calculated

Our web collection on statistics for biologists contains articles on many of the points above.

Software and code

Policy information about availability of computer code

Data collection	NO software was used for data collected
Data analysis	We used GraphPad Prism for graphing and statistical analyses

For manuscripts utilizing custom algorithms or software that are central to the research but not yet described in published literature, software must be made available to editors and reviewers. We strongly encourage code deposition in a community repository (e.g. GitHub). See the Nature Research guidelines for submitting code & software for further information.

Data

Policy information about availability of data

All manuscripts must include a data availability statement. This statement should provide the following information, where applicable:
- Accession codes, unique identifiers, or web links for publicly available datasets
- A list of figures that have associated raw data
- A description of any restrictions on data availability

The data sets generated during and/or analysed during the current study are available from the corresponding author on reasonable request

Field-specific reporting

Life sciences study design

All studies must disclose on these points even when the disclosure is negative.

Sample size	Sample sizes were determined based on previous publications and based on toxicological or physiological standards for statistical anlayses
Data exclusions	We only excluded data that occured due to the lack of mosquito feeding or lack tissue death, which was identified through the fluorophore, Rhodamine B
Replication	All experiments were replicated at least 3 times on different cohorts of mosquiteos to ensure batch variability was accounted for
Randomization	The mosquiotes were selected at random and treated at random
Blinding	The PI coded the chemicals numbers that was not related to their actual names and these numbers changed by the PI randomly to remove bias.

Reporting for specific materials, systems and methods

We require information from authors about some types of materials, experimental systems and methods used in many studies. Here, indicate whether each material, system or method listed is relevant to your study. If you are not sure if a list item applies to your research, read the appropriate section before selecting a response.

Materials & experimental systems

n/a	Involved in the study	
☒	☐	Antibodies
☒	☐	Eukaryotic cell lines
☒	☐	Palaeontology and archaeology
☐	☒	Animals and other organisms
☒	☐	Human research participants
☒	☐	Clinical data
☒	☐	Dual use research of concern

Methods

n/a	Involved in the study	
☒	☐	ChIP-seq
☒	☐	Flow cytometry
☒	☐	MRI-based neuroimaging

Animals and other organisms

Policy information about studies involving animals; ARRIVE guidelines recommended for reporting animal research

Laboratory animals	Aedes aegypti, females used, Paea and Rockafellar strains used
Wild animals	N/A
Field-collected samples	N/A
Ethics oversight	NA

Note that full information on the approval of the study protocol must also be provided in the manuscript.
